# Characteristics of E-cigarette, or Vaping, Products Used by Patients with Associated Lung Injury and Products Seized by Law Enforcement — Minnesota, 2018 and 2019

**DOI:** 10.15585/mmwr.mm6847e1

**Published:** 2019-11-29

**Authors:** Joanne Taylor, Terra Wiens, Jason Peterson, Stefan Saravia, Mark Lunda, Kaila Hanson, Matt Wogen, Paige D’Heilly, Jamie Margetta, Maria Bye, Cory Cole, Erica Mumm, Lauren Schwerzler, Roon Makhtal, Richard Danila, Ruth Lynfield, Stacy Holzbauer, Lung Injury Response Task Force, Lung Injury Response Task Force, Benjamin C. Blount, Mateusz P. Karwowski, Maria Morel-Espinosa, Liza Valentin-Blasini

**Affiliations:** ^1^Epidemic Intelligence Service, CDC; ^2^Minnesota Department of Health; ^3^Council of State and Territorial Epidemiologists Fellowship Program, Atlanta, Georgia; ^4^Center for Preparedness and Response, CDC.; Division of Laboratory Sciences, National Center for Environmental Health, CDC; Division of Laboratory Sciences, National Center for Environmental Health, CDC; Division of Laboratory Sciences, National Center for Environmental Health, CDC; Division of Laboratory Sciences, National Center for Environmental Health, CDC.

During August 9–October 31, 2019, 96 patients were classified as having e-cigarette, or vaping, product use–associated lung injury (EVALI) by the Minnesota Department of Health (MDH); other patients are being investigated for case classification and exposures. Among 58 patients interviewed, 53 (91%) reported obtaining tetrahydrocannabinol (THC)–containing products from informal sources such as friends, family members, or in-person or online dealers. Using gas chromatography–mass spectrometry (GCMS), the MDH Public Health Laboratory (PHL) analyzed 46 THC-containing e-cigarette, or vaping, products obtained from 12 EVALI patients for various potential toxicants, including vitamin E acetate, which has recently been detected in some THC-containing products and in samples of lung fluid from EVALI patients (*1*–*4*). To explore whether vitamin E acetate is a recently added component in THC-containing products, MDH tested ten products seized by law enforcement in 2018, before the EVALI outbreak, and 20 products seized in 2019, during the outbreak. Twenty-four products obtained from 11 EVALI patients from 2019 contained vitamin E acetate. Among the seized products tested by MDH, none seized in 2018 contained vitamin E acetate, although all tested THC-containing products seized in 2019 tested positive for vitamin E acetate. These chemical analyses of products obtained from EVALI patients and of products intended for the illicit market both before and during the outbreak support a potential role for vitamin E acetate in the EVALI outbreak; however, the number of products tested was small, and further research is needed to establish a causal link between exposure to inhaled vitamin E acetate and EVALI. Collaboration between public health jurisdictions and law enforcement to characterize THC-containing products circulating before the recognition of the EVALI outbreak and during the outbreak might provide valuable information about a dynamic market. These Minnesota findings highlight concerns about e-cigarette, or vaping, products that contain THC acquired from informal sources. Because local supply chains and policy environments vary, CDC continues to recommend not using e-cigarette, or vaping, products that contain THC or any e-cigarette, or vaping, products obtained from informal sources. E-cigarette, or vaping, products should never be used by youths, young adults, or pregnant women.* Until the relationship between inhaled vitamin E acetate and lung health is better characterized, vitamin E acetate should not be added to e-cigarette, or vaping, products.

On August 12, the Minnesota Commissioner of Health requested that patients with EVALI be reported. Medical records of suspected cases were reviewed, and patients were classified using CDC case definitions.^†^ EVALI patients or their proxies (e.g., parents) were interviewed using an adaptation of a structured questionnaire developed in Illinois and Wisconsin in consultation with CDC during investigation of cases in those states. Patients were asked to provide product samples to MDH for testing. In addition, to explore whether the content of the local supply of illicit e-cigarette, or vaping, products was different before the outbreak, local law enforcement provided products to MDH from a raid of unregulated manufacturers and distributors of e-cigarette, or vaping, products in 2018 and a comparison sample of products from a raid in 2019 that coincided with the current outbreak.

Product samples from EVALI patients and from the two law enforcement seizures were analyzed at MDH PHL using internally developed headspace GCMS and nontargeted GCMS methods and purchased reference materials. MDH PHL tested for active compounds (cannabidiol [CBD], nicotine, and THC), toxicants of concern (glycerin, medium-chain triglyceride [MCT], propylene glycol, and vitamin E acetate), and three vitamin E forms (alpha, beta, and gamma tocopherol).

Bronchoalveolar lavage (BAL) fluid samples from five EVALI patients were analyzed at CDC for active compounds and toxicants. MDH collaborated with the Minnesota Bureau of Criminal Apprehension Forensic Drug Chemistry Department, which had obtained six containers of bulk liquids, each labeled with a different flavor, and 100 cartridges (all labeled “Cali Plugs Grape Punch”) from a spring 2018 raid. PHL tested five bulk liquid samples and five cartridges held by the Minnesota Bureau of Criminal Apprehension. In September 2019, local law enforcement seized 75,000 cartridges intended for the illicit THC market (*5*). These cartridges were packaged inside boxes bearing two market labels: “Dank Vapes” and “31 Flavors.” Investigators used labeling to identify 31 different flavors of Dank Vapes and 19 different flavors of 31 Flavors. PHL evaluated 10 different flavor cartridges labeled “Dank Vapes” and 10 different flavor cartridges labeled “31 Flavors.”

As of October 31, 2019, 96 patients were classified as having confirmed or probable EVALI in Minnesota, and additional cases are being investigated. The median age of patients was 21 years (range = 15–71 years), and 58 (60%) were male. Eighty-seven (91%) patients were hospitalized, including 26 (27%) in intensive care units. Three (3%) patients died. Among 58 (60%) interviewed EVALI patients, 53 (91%) reported using illicit THC-containing products obtained from informal sources in the 3 months before illness onset,^§^ 41 (71%) used nicotine-containing products, and 14 (24%) used CBD oil products (Table). Two patients reported using illicit THC, medical cannabis, and nicotine-containing products. Thirty-nine (67%) patients reported using Dank Vapes.

**TABLE:** E-cigarette, or vaping, product use characteristics of interviewed e-cigarette, or vaping, product use–associated lung injury (EVALI) patients (N = 58) — Minnesota, 2019

Product use characteristics (no. with available information if <58)	No. (%)
**Illicit THC-containing products**	
Any use*	53 (91)
Exclusive use	13 (22)
Prefilled cartridges^†^	47 (81)
**Nicotine-containing products**	
Any use	41 (71)
Exclusive use	2 (3)
Nicotine use, without illicit THC	3 (5)
**Any use, both illicit THC- and nicotine-containing products**	37 (64)
**Both illicit THC- and nicotine-containing products only**	26 (45)
**CBD-containing products**	
Any use	14 (24)
CBD oil products, with illicit THC and nicotine	8 (14)
CBD oil with illicit THC	3 (5)
CBD and nicotine	1 (2)
**Other product combinations^§^**	
**Illicit THC brand usage**	
Any use Dank Vapes^¶^	39 (67)
Used Dank Vapes exclusively, with no other THC brands	11 (19)
Did not use Dank Vapes, but used other THC brands**	6 (10)
Solely used Dank Vapes, no other THC brand, nicotine, or CBD oil	2 (3)
**Illicit THC- and nicotine-containing product use frequency^††^**	
Daily use of THC-containing products (49)	37 (76)
Daily use of nicotine-containing products (40)	32 (80)
**Illicit THC- and nicotine-containing product use duration^††^**	
>1-year use of THC-containing products (37)	19 (51)
>1-year use of nicotine-containing products (31)	22 (71)

**Abbreviations:** CBD = cannabidiol; THC = tetrahydrocannabinol.

* Three patients reported use of CBD and THC only. Two additional patients did not report THC during interview, but testing of bronchoalveolar lavage fluid or product confirmed exposure to THC.

^†^ Prefilled cartridge use was unknown for six respondents who used illicit THC.

^§^ Nicotine and unknown homemade oil (one, laboratory testing confirmed THC and vitamin E acetate present in bronchoalveolar lavage fluid); CBD and unknown (one, laboratory testing of product confirmed THC and vitamin E acetate in unlabeled cartridge); and illicit THC, nicotine, and prescribed THC products supplied by a medical marijuana dispensary (two).

^¶^ Dank Vapes are a class of largely counterfeit THC-containing products of unknown provenance that are marketed under a common name and distributed through informal sources.

** EVALI patients reported using these non-Dank Vapes brands: Banks Extracts (one), Cannaclear (one), Chronic (one), Cookie Cart (one), Dabwoods (one), King Pin (one), Off White (two), Runtz (one), Sauce Extracts (one), TKO Extracts (two), and West Coast Cure (one). Cartridge or brand use was unknown for eight respondents who used illicit THC.

^††^ Information on brand of THC-containing product and THC use duration and frequency was not available for all patients.

Sixteen (28%) patients submitted 265 products, 67 of which were selected for testing because of available product volume and features that physically differentiated the cartridges; 46 contained THC, and 21 contained nicotine. Among the 46 assessed THC-containing products submitted by 12 patients, the most commonly detected compounds were vitamin E acetate (24, 52%), MCT (20, 43%), CBD (20, 43%), and alpha tocopherol (17, 37%). Eight (17%) THC-containing products did not contain either vitamin E acetate or MCT. THC-containing products used by 11 of 12 (92%) patients contained vitamin E acetate, and products from seven (58%) patients contained MCT. One patient who used medical cannabis submitted illicit THC-containing products; one tested product contained vitamin E acetate and another contained MCT. THC-containing products from one patient did not contain vitamin E acetate; however, this patient reported using multiple products daily, including Dank Vapes, which were not included among the products submitted for testing. Among 21 nicotine-containing products submitted by eight patients, 20 contained propylene glycol, and 15 contained glycerin but not the other analytes.

Among the 21 patient-submitted THC-containing products that were categorized by identifiable brands, two of two Dank Vapes samples contained vitamin E acetate (Figure). In five products labeled “Dr. Zodiak” and six labeled “TKO Extract,” vitamin E acetate, MCT, and alpha tocopherol were variably detected.

**FIGURE:**
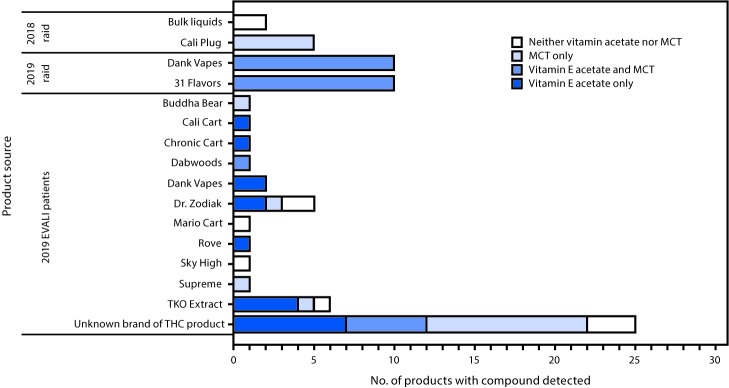
Detection of vitamin E acetate and medium chain triglyceride (MCT) by mass spectrometry methods in tetrahydrocannabinol (THC)–containing products obtained from e-cigarette, or vaping, product use–associated lung injury patients (N = 46) and law enforcement raids (N = 27) — Minnesota, 2018 and 2019

Vitamin E acetate was detected in all five patient BAL fluid specimens. One of these patients submitted four THC-containing cartridges labeled “TKO Extract” (two), “Rove” (one), or “Dr. Zodiak” (one), all of which contained vitamin E acetate. Although the other patients were known to be exposed to THC based on interview or testing of the BAL fluid, none of the other patients whose BAL fluid specimens were tested submitted THC-containing products for testing.

Among products seized during the 2018 raid, all five bulk liquid samples tested negative for vitamin E acetate and MCT; two tested positive for THC (Figure). The bulk liquids appeared to be flavoring agents. All five Cali Plug cartridges tested contained THC and MCT, but not vitamin E acetate. Among the 20 tested cartridges seized during September 2019, all contained THC, vitamin E acetate, and MCT. In addition, five cartridges of 31 Flavors also contained gamma tocopherol.

## Discussion

This report evaluated e-cigarette, or vaping, products used by EVALI patients and products intended for the illicit market and seized by law enforcement both before and during the current EVALI outbreak. The findings support a potential role for vitamin E acetate in lung injury in EVALI patients. Vitamin E acetate has been detected in a high proportion of THC-containing products associated with EVALI cases, including those tested at MDH PHL, New York (*4*), Utah (*3*), and the Food and Drug Administration (from 25 states) (*2*). In addition, evaluation at CDC of 29 BAL fluid specimens from a convenience sample of EVALI patients from 10 states, including five Minnesota patients, found vitamin E acetate in all specimens (*1*). Although the long-term stability of vitamin E acetate in THC-containing products is unknown, vitamin E acetate is reported to remain stable for at least 36 months in cosmetic products (*6*). Whereas vitamin E acetate was not detected in the limited number of tested products seized in 2018, it was detected in products seized in 2019, suggesting that vitamin E acetate might have been introduced recently as a diluent or filler. However, verification of this observation requires testing of more products from Minnesota before 2019 as well as products from other states.

All Dank Vapes tested, including those from patients and those confiscated by law enforcement in 2019, contained vitamin E acetate. The majority of interviewed EVALI patients in Minnesota reported using Dank Vapes products: to date, 87%–95% of EVALI patients in Illinois, Minnesota, Utah, and Wisconsin have reported using illicit THC-containing products, with 40%–75% of patients interviewed reporting using cartridges labeled “Dank Vapes” (*3*,*7*). In Illinois, EVALI patients aged 18–44 years had higher odds of reporting use of illicit THC-containing products and of using products labeled “Dank Vapes,” compared with persons aged 18–44 years who in an online survey reported use of THC-containing products but who did not have EVALI (*8*).

MCT was found in many of the THC-containing products tested in Minnesota. Although the numbers are small, MCT was not found in any of the 29 tested BAL fluid samples from EVALI patients (*1*). MCT was found in products seized by law enforcement during both 2018 and 2019, suggesting that persons using illicit THC-containing products in Minnesota were exposed to MCT before 2019. However, more information on MCT is needed. Alpha tocopherol and gamma tocopherol were detected in some products. These forms of vitamin E can be naturally derived from plant products; whether these compounds have a role in EVALI is unknown (*9*). Additional work, including quantitative analysis of the various compounds in products, assessment of interactions and changes occurring with heating, and assessment of the biologic activity of potential toxicants in animal models should be considered.

Because many EVALI patients used THC- and nicotine-containing products, nicotine-containing products were evaluated as well. None of the nicotine-containing products tested contained alpha tocopherol, gamma tocopherol, MCT, THC, or vitamin E acetate.

Two EVALI-patients discussed here used medical cannabis vaping products as well as illicit THC-containing e-cigarette, or vaping, products. One patient submitted illicit THC-containing e-cigarette, or vaping, products; one product tested contained vitamin E acetate and another contained MCT. Another EVALI-patient refused interview, but medical records indicated that the patient was enrolled in the medical cannabis program and also used illicit THC-containing e-cigarette, or vaping, products. After the analytic period covered by this report, MDH learned of two additional patients who used medical cannabis products, one of whom reported use of marijuana. The type of medical cannabis used by these patients, as analyzed by MDH PHL, does not contain vitamin E acetate or MCT. Another medical cannabis manufacturer in Minnesota had used MCT, but no longer uses this compound. Further investigation of these patients is ongoing.

The findings in this report are subject to at least six limitations. First, EVALI patients might have been misclassified. Second, many EVALI patients did not agree to be interviewed or to provide products for testing, which might limit the generalizability of these findings to other EVALI patients. Third, products submitted by EVALI patients did not represent all THC- and nicotine-containing products they had recently used. Fourth, many products did not contain sufficient material to test. Fifth, mass spectrometric laboratory testing of products focused on 10 compounds for which reference materials were obtained; however, other toxicants might have been present but not identified. Finally, only a limited number of products and brands from law enforcement were tested, and these might not be representative of available products in Minnesota.

Although vitamin E acetate was detected in THC-containing products provided by 11 of 12 EVALI patients and a convenience sample of confiscated products from 2019 in Minnesota, additional analyses are needed to establish whether a causal link exists between inhaled vitamin E acetate exposure and EVALI. According to these and other published data, using THC-containing products with vitamin E acetate appears to be associated with EVALI; however, it is possible that more than one compound or ingredient could be a cause of lung injury, and evidence is not yet sufficient to rule out contribution of other toxicants. The ongoing investigation in Minnesota has shown that collaborating with law enforcement to obtain and test products confiscated before and during the current outbreak can provide valuable information on the potential changes in these products in a dynamic market. Such collaboration is encouraged elsewhere to provide insight into the national picture and an improved understanding of THC-containing products. These findings from Minnesota highlight concerns about e-cigarette, or vaping, products that contain THC acquired from informal sources such as friends, family members, or in-person or online dealers. Because local supply chains and policy environments vary, CDC continues to recommend not to use e-cigarette, or vaping, products that contain THC and not to use any e-cigarette, or vaping, products obtained from informal sources. Further, e-cigarette, or vaping, products should never be used by youths, young adults, or pregnant women. Until the relationship between vitamin E acetate and lung health is better characterized, vitamin E acetate should not be added to e-cigarette, or vaping, products.

**Summary**

**What is already known about this topic?**

Tetrahydrocannabinol (THC)-containing e-cigarette, or vaping, products also containing vitamin E acetate appear to be associated with e-cigarette, or vaping, product use–associated lung injury (EVALI).

**What is added by this report?**

Illicit THC-containing products submitted by 11 of 12 EVALI patients in Minnesota contained vitamin E acetate. Twenty THC-containing products seized during September 2019 contained vitamin E acetate; ten products seized during 2018, before the EVALI outbreak, did not contain vitamin E acetate.

**What are the implications for public health practice?**

These data further support a potential role for vitamin E acetate in EVALI. While potential toxicants continue to be evaluated, vitamin E acetate should not be added to e-cigarette, or vaping, products.

## Footnotes

* https://e-cigarettes.surgeongeneral.gov/documents/surgeon-generals-advisory-on-e-cigarette-use-among-youth-2018.pdf.

† https://www.cdc.gov/tobacco/basic_information/e-cigarettes/assets/2019-Lung-Injury-Surveillance-Case-Definition-508.pdf.

§ THC-containing products were obtained from informal sources such as friends, family members, or in-person or online dealers, and were not obtained from the Minnesota medical cannabis program (https://www.health.state.mn.us/people/cannabis/).
